# Early pulmonary hypertension is a risk factor for bronchopulmonary dysplasia-associated late pulmonary hypertension in extremely preterm infants

**DOI:** 10.1038/s41598-021-90769-4

**Published:** 2021-05-27

**Authors:** Hyun Ho Kim, Se In Sung, Mi Sun Yang, Yea Seul Han, Hye Seon Kim, So Yoon Ahn, Ga Won Jeon, Yun Sil Chang, Won Soon Park

**Affiliations:** 1 Research Institute of Clinical Medicine of Jeonbuk National University, Jeonju, South Korea; 2 Biomedical Research Institute of Jeonbuk National University Hospital, Jeonju, South Korea; 3 Department of Pediatrics, Samsung Medical Center, Sungkyunkwan University School of Medicine, Seoul, South Korea; 4 Department of Pediatrics, Hallym University Kangnam Sacred Heart Hospital, Seoul, South Korea; 5 Department of Pediatrics, Busan Paik Hospital, Inje University College of Medicine, Busan, South Korea

**Keywords:** Medical research, Paediatric research

## Abstract

This study evaluated whether early pulmonary hypertension (PH) in extremely preterm infants (EPIs) at 22–27 weeks of gestation detected clinically with echocardiography at 4–7 postnatal days (PND) is a risk factor for death before 36 weeks post-menstrual age (PMA) or late PH in moderate or severe (m/s) bronchopulmonary dysplasia (BPD) (BPD-PH). We analyzed risk factors for death before 36 weeks PMA or BPD-PH. Among 247 EPIs enrolled, 74 (30.0%) had early PH. Twenty-one (28.4%) infants with early PH and 18 (10.4%) without early PH died before 36 weeks PMA; 14 (18.9%) infants with early PH and 9 (5.2%) without early PH had BPD-PH at 36–38 weeks PMA. Multivariate analysis revealed that early PH (adjusted odds ratio, 6.55; 95% confidence interval, 3.10–13.82, *P* < 0.05), clinical chorioamnionitis (2.50; 1.18–5.31), intraventricular hemorrhage (grade 3–4) (3.43; 1.26–9.37), and late sepsis (6.76; 3.20–14.28) independently increased the risk of development of death before 36 weeks PMA or BPD-PH. Subgroup analysis among m/s BPD patients revealed that early PH (4.50; 1.61–12.58) and prolonged invasive ventilator care (> 28 days) (4.91; 1.02–23.68) increased the risk for late PH independently. In conclusion, EPIs with early PH at 4–7 PND should be monitored for BPD-associated late PH development.

## Introduction

Pulmonary hypertension (PH) is associated with significant mortality and morbidity in extremely preterm infants (EPIs). PH in EPIs presents in two phases as “early” and “late”. Early PH in preterm infants occurs in the early period after birth, with similar clinical or echocardiographic manifestations of persistent pulmonary hypertension of the newborn (PPHN) in term infants, especially with prolonged maternal preterm premature rupture of the membranes (PPROM) and oligohydramnios^1^. Early PH in preterm infants is a significant risk factor for bronchopulmonary dysplasia (BPD) and death^1–4^. In contrast, late PH occurring later in preterm infants is recently recognized as mostly related to BPD. BPD is known to be the strongest factor associated with the development of late PH. Moreover, mortality is significantly higher in infants with late PH than in infants without^5^. Therefore, better strategies are needed to improve survival in this vulnerable group of preterm infants.

To develop such strategies, better insight into the occurrence of PH in EPIs, including identification of risk factors, is strongly required. Several factors, such as oligohydramnios or intrauterine growth retardation, are well-known risk factors for early PH. However, risk factors for late PH overlap with those for BPD, and there are no known BPD-specific risk factors^6^. Moreover, whether early PH is associated with and thus predictive of late PH, especially when associated with BPD in preterm infants, is inconclusive. Previously, Mourani et al. have prospectively demonstrated that early vascular maladaptation showing early PH identified at 7 days of age by echocardiography in very low birthweight (VLBW) infants was found to be a significant risk factor for subsequent development of late PH regardless of BPD at 36 weeks’ postmenstrual age (PMA)^3^. However, studies conducted in EPIs below 28 weeks’ gestation at high risk for BPD did not show a significant association between early and late PH in prospective^2,7^ or retrospective studies^8^. Therefore, the association between early and late PH in EPIs at risk for BPD needs to be further clarified.

Recently, an initial echocardiogram within 1 week after birth is frequently conducted for the care of EPIs in neonatal intensive care units (NICUs), which in turn has a better chance of detecting signs of early PH^9^. On the other hand, echocardiographic screening for late PH after 36 weeks PMA for confirmed BPD patients in the NICU with an emphasis on the increased risk for late PH in established BPD infants is becoming a common clinical practice in NICUs^10,11^. In these clinical settings, if early PH detected by early echocardiography during the first week of life is confirmed as a significant risk factor for the development of late PH associated with BPD, appropriate strategies for the screening, prevention, and treatment of late PH occurring in BPD patients (BPD-PH) can be developed for EPIs at high risk of BPD.

Therefore, this study aimed to evaluate whether early PH clinically detected by echocardiography performed at 4–7 postnatal days (PND) after a transitional period is an independent risk factor for the development of late PH detected by echocardiographic screening in moderate or severe (m/s) BPD infants (BPD-PH) as well as death before 36 weeks PMA, which is a competing outcome for BPD-PH in EPIs born at < 28 weeks of gestation.

## Results

### Population

**Figure 1:**
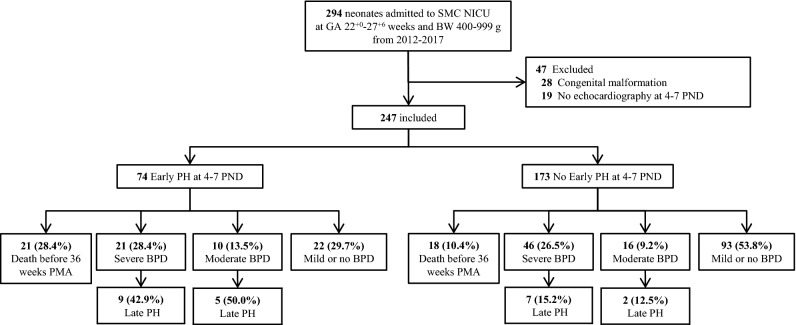
The study population. *SMC* Samsung Medical Center, *NICU* neonatal intensive care unit, *GA* gestational age, *BW* birthweight, *PND* postnatal days, *PH* pulmonary hypertension, *BPD* bronchopulmonary dysplasia.

A total of 294 infants with a gestational age of 22–27 weeks and birth weight of 401–999 g were delivered at and admitted to Samsung Medical Center between 2012 and 2017. After excluding 28 infants with major congenital malformations and 19 infants with absent echocardiographic data on 4–7 PND, 247 EPIs were included in this study (Fig. 1).

Among them, 74 infants (30.0%) were diagnosed with early PH, and 173 (70.0%) did not have early PH. Twenty-one (28.4%) infants with early PH and 18 (10.4%) without early PH died before 36 weeks PMA; 31 (41.9%) infants with early PH and 62 (35.8%) infants without early PH were diagnosed with m/s BPD at 36 weeks PMA; and 14 (18.9%) infants with early PH and 9 (5.2%) without early PH were diagnosed with late PH associated with m/s BPD (BPD-PH) at 36–38 weeks PMA, with an incidence of 45.2% and 14.5%, respectively, among the subgroups of m/s BPD infants. Among the 23 infants with late PH with m/s BPD, 14 (61%) had early PH, whereas among 70 infants with m/s BPD without late PH, 17 (24%) had early PH. When stratified by BPD severity, 16 (23.9%) infants with severe BPD (n = 67) and 7 (26.9%) infants with moderate BPD (n = 26) were diagnosed with late PH.

PMA or PND at echocardiography was not different between infants with or without early PH and infants with or without BPD-PH (Table 1). The average timing of the early echocardiography was 5.8 ± 1.1 PND. In detail, 40 (16.2%) EPIs received early echocardiography on PND 4, 82 (33.2%) on 5, 83 (33.6%) on 6, and 42 (17.0%) on 7. The most frequently observed echocardiographic findings of PH were ventricular septal flattening or a D-shaped left ventricle, followed by increased pulmonary artery pressure (> 40 mmHg) as estimated by peak Doppler velocity of tricuspid regurgitation (Supplementary Table 1).

**Table 1:** Perinatal demographic characteristics.

	No Early PH (N = 173)	Early PH (N = 74)	*P* value	No death before 36 weeks PMA or BPD-PH (N = 185)	Death before 36 weeks PMA or BPD-PH (N = 62)	*P* value
**Neonatal characteristics**						
Age at echocardiography, postnatal days	5.9 ± 1.0	5.7 ± 1.2	0.23	84.1 ± 10.9*	85.1 ± 10.5*	0.70
PMA at echocardiography, weeks	26.2 ± 1.2	26.0 ± 1.2	0.10	36.9 ± 0.7*	37.2 ± 0.9*	0.18
Gestational age, (week)	25.2 ± 1.3	25.2 ± 1.3	0.76	25.3 ± 1.2	24.8 ± 1.4	< 0.05
22 + 0 to 23 + 6 weeks, n (%)	31 (17.9)	12 (16.2)	0.96	26 (14.1)	17 (27.4)	< 0.05
24 + 0 to 25 + 6 weeks, n (%)	86 (49.7)	44 (59.5)	0.16	89 (53.0)	32 (51.6)	0.85
26 + 0 to 27 + 6 weeks, n (%)	56 (32.4)	18 (24.3)	0.21	61 (33.0)	13(21.0)	0.07
Birthweight (g)	722.5 ± 137.7	705.1 ± 154.2	0.38	734.8 ± 134.1	665.2 ± 155.7	< 0.05
400–600 g, n (%)	31 (16.8)	18 (24.3)	0.25	28 (15.1)	19 (30.6)	< 0.05
600–800 g, n (%)	86 (49.1)	33 (44.6)	0.46	89 (48.1)	29 (46.8)	0.86
800–1000 g, n (%)	56 (34.1)	23 (31.1)	0.60	68 (36.8)	14 (22.6)	< 0.05
Small for gestational age, n (%)	28 (16.2)	18 (24.3)	0.13	30 (16.2)	16 (25.8)	0.09
Male, n (%)	79 (45.7)	45 (60.8)	< 0.05	89 (48.1)	35 (56.5)	0.26
1-min Apgar score	4.6 ± 1.4	4.2 ± 1.4	0.05	4.6 ± 1.4	4.2 ± 1.6	0.07
5-min Apgar score	7.1 ± 1.3	6.7 ± 1.7	0.05	7.0 ± 1.4	6.9 ± 1.4	0.09
**Maternal characteristics**						
Maternal age, year	33.3 ± 3.5	32.9 ± 4.2	0.47	33.1 ± 3.8	33.4 ± 3.4	0.57
Multiple gestation, n (%)	63 (36.4)	23 (31.1)	0.42	66 (35.7)	20 (32.3)	0.63
Pregnancy induced hypertension, n (%)	17 (9.8)	11 (14.9)	0.25	23 (12.4)	5 (8.1)	0.35
Oligohydramnios, n (%)	42 (24.3)	28 (37.8)	< 0.05	45 (24.3)	25 (40.3)	< 0.05
In vitro fertilization, n (%)	50 (28.9)	23 (31.1)	0.73	54 (29.2)	19 (30.6)	0.83
Clinical chorioamnionitis, n (%)	95 (54.9)	44 (59.5)	0.51	94 (50.8)	45 (72.6)	< 0.05
Pathologic chorioamnionitis, n (%)	119 (68.8)	42 (56.8)	0.07	114 (61.6)	47 (75.8)	< 0.05
Cesarean section, n (%)	127 (73.4)	53 (71.6)	0.77	142 (76.8)	38 (61.3)	< 0.05
Gestational diabetes, n (%)	13 (7.5)	1 (1.4)	0.07	10 (5.4)	4 (6.5)	0.76
Complete antenatal steroid, n (%)	113 (65.3)	65 (87.8)	0.64	169 (91.4)	58 (93.5)	0.58
Duration of PPROM, day	3.8 ± 7.4	5.4 ± 10.1	0.25	3.7 ± 7.7	6.2 ± 10.0	0.73
PPROM duration ≥ 28 days, n (%)	3 (1.7)	6 (8.1)	< 0.05	3 (1.6)	5 (8.1)	< 0.05
PPROM at < 26 weeks’ gestation, n (%)	18 (5.2)	9 (23.0)	< 0.05	23 (12.4)	12 (19.4)	0.18

*Echocardiographies were done only in m/s BPD patients at 36–38 weeks PMA; *PH* pulmonary hypertension, *m/s BPD* moderate to severe bronchopulmonary dysplasia, *BPD-PH* m/s BPD associated late PH, *PPROM* preterm premature rupture of membranes, *PMA* post-menstrual age. Data are show as N (%) or mean ± SD.

### Demographic characteristics

Perinatal demographic characteristics were compared between infants with and without early PH as well as between infants with and without the primary composite outcome of death before 36 weeks PMA or BPD-PH. Average gestational age, birthweight, and other perinatal characteristics were not significantly different between infants with and without early PH. However, infants with early PH had a higher incidence of male sex, maternal oligohydramnios, prolonged premature rupture of membrane (PPROM) ≥ 28 days, and PPROM at < 26 weeks’ gestation than infants without early PH.

When we compared infants with and without the primary outcome of death before 36 weeks PMA or BPD-PH, those with the primary outcome had lower gestational age and birth weight, higher incidence of oligohydramnios, clinical or pathologic chorioamnionitis, PPROM at ≥ 28 days, and lower incidence of cesarean section (Table 1).

When comparing the subgroup of m/s BPD infants with and without late PH, all variables were similar except for the incidences of small for gestational age (SGA) and maternal prolonged PPROM (duration ≥ 28 days), which were higher in infants with late PH than in infants without (Supplementary Table 2).

### Neonatal outcomes

Infants with early PH showed significantly higher incidences of m/s BPD and in-hospital mortality, especially within 1 month of age, than infants without early PH; the causes of in-hospital death are listed in Supplementary Table 3. Sepsis (33.3%) was the most common cause of death, followed by necrotizing enterocolitis (NEC; 30.8%) and pulmonary hypoplasia (10.3%). Pulmonary hypoplasia was a more frequent cause of death in infants with early PH than in those without.

Infants with the primary outcome of death before 36 weeks PMA or BPD-PH had a higher incidence of early PH, severe intraventricular hemorrhage (IVH; ≥ grade 3), prolonged invasive ventilation (> 28 days), early sepsis, late sepsis before 36 weeks PMA, and death after 36 weeks PMA (Table 2). Meanwhile, among the subgroup of m/s BPD infants, the incidence of early PH, prolonged invasive ventilation (> 28 days), and death after 36 weeks PMA was significantly higher in infants with late PH than in infants without (Supplementary Table 4).

**Table 2:** Neonatal morbidities and mortality.

	No early PH (N = 173)	Early PH (N = 74)	*P* value	No death before 36 weeks PMA or BPD-PH (N = 185)	Death before 36 weeks PMA or BPD-PH (N = 62)	*P* value
Early PH, n (%)	–	–	–	39 (21.1)	35 (56.5)	< 0.05
IVH grade 3–4, n (%)	20 (11.6)	10 (13.5)	0.67	17 (9.2)	13 (21.0)	< 0.05
PDA > 2 mm (%) at early echocardiogram*	106 (61.3)	51 (68.9)	0.25	119 (64.3)	38 (61.3)	0.67
Enrollment for PDA RCT^23^ (%)	42 (24.3)	15 (20.3)	0.50	45 (24.3)	12 (19.4)	0.42
m/s BPD or death (before 36 weeks PMA), n (%)	80 (46.0)	42 (70.0)	< 0.05	–	–	–
NEC grade 2b-3, n (%)	15 (8.7)	5 (6.8)	0.71	7.6 (14)	6 (9.7)	0.60
Invasive ventilator before 36 weeks PMA, day	31.3 ± 22.2	32.8 ± 21.2	0.62	29.6 ± 21.3	37.9 ± 22.7	< 0.05
Invasive ventilator > 28 days before 36 weeks PMA, n (%)	79 (45.7)	38 (51.4)	0.41	77 (41.6)	40 (64.5)	< 0.05
Early sepsis (< 7 days), n (%)	8 (4.6)	5 (6.8)	0.54	5 (2.7)	8 (12.9)	< 0.05
Late sepsis (7 days–36 weeks PMA), n (%)	52 (30.1)	21 (28.4)	0.79	39 (21.1)	34 (54.8)	< 0.05
ROP stage 3–4, n (%)	50 (28.9)	28 (37.8)	0.17	63 (34.1)	15 (24.2)	0.15
Periventricular leukomalacia, n (%)	15 (9.0)	4 (5.0)	0.38	14 (7.6)	5 (8.1)	1.00
**Death at discharge, n (%)**	25 (14.5)	22 (29.7)	< 0.05	4 (2.2)	44 (71.0)	< 0.05
Death at before 1 month, n (%)	6 (3.5)	12 (16.2)	< 0.05	0 (0.0)	18 (29.0)	< 0.05
Death at 1 month–36 weeks PMA, n (%)	12 (6.9)	8 (10.8)	0.31	0 (0.0)	18 (29.0)	< 0.05
Death after 36 weeks PMA, n (%)	7 (4.0)	2 (2.8)	0.61	4 (2.2)	8 (12.9)	< 0.05
**Support at the time of echocardiogram**						
HFOV or conventional ventilator, n (%)^†^	134 (77.5)	70 (94.6)	< 0.05	6/70 (8.6)	5/23 (21.7)	0.13
BiPAP or CPAP, n (%)^†^	29 (16.8)	4 (5.4)	< 0.05	27/70 (38.6)	8/23 (34.8)	0.75
HFNC, n (%)^†^	7 (4.0)	0 (0.0)	0.11	8/70 (11.4)	3/23 (13.0)	1.00
Nasal canula, n (%)^†^	0 (0.0)	0 (0.0)	–	29/70 (41.4)	7/23 (30.4)	0.35
FiO_2_, %^†^	23.2 ± 5.6	28.8 ± 17.3	< 0.05	25.4 ± 5.4	25.2 ± 4.4	0.91
Inhaled Nitric Oxide, n (%)^†^	0 (0.0)	5 (6.8)	–	0/70 (0.0)	0/23 (0.0)	–
Sildenafil or other pulmonary vasodilators, n (%)^†^	0 (0.0)	0 (0.0)	–	0/70 (0.0)	0/23 (0.0)	–

*PDA was managed using conventional nonintervention treatment (not to treat) with careful fluid management based on our unit policy^22^.

^†^Calculated only for infants with an early or late echocardiogram, *PH* pulmonary hypertension, *m/s BPD* moderate to severe bronchopulmonary dysplasia, *BPD-PH* m/s BPD associated late PH, *IVH* intraventricular hemorrhage, *RCT* randomized clinical study, *NEC* necrotizing enterocolitis, *PMA* post-menstrual age, *ROP* retinopathy of premature, *HFOV* high frequency oscillatory ventilation, *BiPAP* bilevel positive airway pressure, *CPAP* continuous positive airway pressure, *PDA* patent ductus arteriosus, *PMA* post-menstrual age. Data are show as N (%) or mean ± SD.

As for respiratory support and pulmonary vasodilator treatment at the time of echocardiography, infants with early PH required a more invasive respiratory support and oxygen treatment at higher concentrations. No differences in respiratory support level were noted when late echocardiography was performed at 36–38 weeks PMA between m/s BPD infants with and without late PH. Five (6.8%) infants with early PH received inhaled nitric oxide therapy at the time of early echocardiography based on the clinical diagnosis of PPHN. No infant with m/s BPD infants received pulmonary vasodilator treatment at the time of late echocardiography (Table 2).

### Risk factor analysis

To identify significant risk factors for the development of the primary outcome of death before 36 weeks PMA or BPD-PH, we performed univariate analysis using perinatal and neonatal factors in all enrolled infants. The significant risk factors were gestational age, cesarean section, oligohydramnios, clinical and pathologic chorioamnionitis, prolonged PPROM (≥ 28 days), early PH, IVH (grade 3–4), early and late sepsis, and prolonged invasive ventilator care (> 28 days). Multivariate analysis using these factors revealed that early PH (adjusted odds ratio [OR], 6.55; 95% confidence interval [CI], 3.10–13.82, *P* < 0.05), clinical chorioamnionitis (adjusted OR, 2.50; 95% CI, 1.18–5.31, *P* < 0.05), IVH (grade 3–4) (adjusted OR, 3.43; 95% CI, 1.26–9.37, *P* < 0.05), and late sepsis (adjusted OR, 6.76; 95% CI, 3.20–14.28, *P* < 0.05) independently increased the risk of development of death before 36 weeks PMA or BPD-PH (Table 3).

**Table 3:** Risk factors associated with primary outcome of death prior to 36 weeks PMA or BPD associated late pulmonary hypertension.

	Unadjusted				Adjusted			
	OR	95% CI		*P* value	OR	95% CI		*P* value
Gestational age, week	0.70	0.55	0.88	< 0.05				
Cesarean section	0.48	0.26	0.89	< 0.05	0.47	0.22	1.02	0.06
Oligohydramnios	2.10	1.14	3.86	< 0.05	1.98	0.94	4.17	0.07
Clinical chorioamnionitis	2.56	1.37	4.80	< 0.05	2.50	1.18	5.31	< 0.05
Pathologic chorioamnionitis	1.95	1.02	3.74	< 0.05				
PPROM duration ≥ 28 days	5.32	1.23	22.96	< 0.05				
Early PH	4.85	2.63	9.00	< 0.05	6.55	3.10	13.82	< 0.05
IVH grade 3–4	2.62	1.19	5.77	< 0.05	3.43	1.26	9.37	< 0.05
Invasive ventilator > 28 days before 36 weeks PMA	2.55	1.40	4.63	< 0.05				
Early sepsis	5.33	1.68	16.98	< 0.05				
Late sepsis	4.55	2.46	8.39	< 0.05	6.76	3.20	14.28	< 0.05

*PH* pulmonary hypertension, *PMA* postmenstrual age, *PPROM* preterm premature rupture of membranes, *IVH* interventricular hemorrhage, *CI* confidence interval.

To identify the risk factors for late PH development in infants with m/s BPD, a subgroup analysis was performed in m/s BPD infants. SGA, prolonged PPROM (≥ 28 days), early PH, and prolonged invasive ventilator care (> 28 days) were significant based on the univariate analysis. Multivariate analysis using these factors revealed that early PH (adjusted odds ratio [OR], 4.50; 95% confidence interval, 1.61–12.58, *P* < 0.05) and prolonged invasive ventilator care (> 28 days) (adjusted OR, 4.91; 95% confidence interval, 1.02–23.68, *P* < 0.05) independently increased the risk for development of late PH in m/s BPD infants (Table 4).

**Table 4:** Risk factors associated with late PH among moderate to severe BPD patients.

	Moderate to severe BPD (N = 93)							
	Unadjusted				Adjusted			
	OR	95% CI		*P* value	OR	95% CI		*P* value
Gestational age, week	1.11	0.76	1.62	0.60				
Small for gestational age	3.11	1.10	8.82	< 0.05				
Early PH	4.85	1.78	13.18	< 0.05	4.50	1.61	12.58	< 0.05
Invasive ventilator > 28 days before 36 weeks PMA	5.48	1.18	25.35	< 0.05	4.91	1.02	23.68	< 0.05
PPROM duration ≥ 28 days	10.35	1.02	105.03	< 0.05				

*PH* pulmonary hypertension, *m/s BPD* moderate to severe bronchopulmonary dysplasia, *PMA* postmenstrual age, *PPROM* preterm premature rupture of membranes, *CI* confidence interval.

## Discussion

In the present study, we demonstrated that early PH, as evidenced by echocardiographic findings on PND 4–7 in EPIs, is an independent risk factor for death before 36 weeks PMA or BPD-PH identified by echocardiographic screening at 36–38 weeks PMA. Furthermore, the present study also showed a strong association between early and late PH in a subset cohort of patients with m/s BPD.

The reported incidence of PH in preterm infants varies between studies over a wide range due to variability in the timing of echocardiographic screening, inclusion criteria, and study settings, as well as variable definitions for PH by clinical or echocardiographic perspectives. Early PH in preterm infants was reported frequently in early life as 42% at 7 days of life among infants with birthweights 500–1250 g by Mourani et al.^3^ and 55% at 72–96 h of life and 38% at 5–14 postnatal days among EPIs (< 29 weeks) by Mirza et al.^4^. However, it was reported as 6.2% at 4–6 weeks postnatally in extremely LBW infants by Bhat et al.^10^. In addition, when the definition of PH was restricted to moderate to severe PH, the incidence of early PH was reported to be much lower even in early life: 8% at 10–14 postnatal days in EPIs (with < 28 weeks’ gestation) by Mirza et al.^2^ and 13.5% at 7 days of life with the alternative stricter definition by Mourani et al.^3^. All four studies were prospectively designed. However, in retrospective studies, it was reported to be slightly lower (22.7%) in VLBW infants at 4–14 days of life^12^ and 20% at 5–14 days in EPIs with 22–28 weeks’ gestation^8^, as diagnosed by clinically indicated echocardiographic evaluation. However, it was much lower (8.1%) in VLBW infants (< 28 weeks’ gestation) in a large retrospective cohort study when early PH was defined as clinical or echocardiographic PPHN^13^. The incidence of early PH in the present study was 30% among included EPIs, which is similar but slightly lower than that of prospectively designed studies. Although we designed this study retrospectively, all infants included received echocardiographic examination on PND 4–7 for variable clinical reasons, which simulates a prospective study, although not focused on early PH itself.

In the present study, the significant risk factors for early PH were male sex, the presence of oligohydramnios, and earlier onset of prolonged PPROM below 26 weeks’ gestation as well as prolonged duration (≥ 28 days) of PPROM, similar to other previous studies^1,14^. This result suggests the importance of possible pulmonary hypoplasia due to sequential PPROM and oligohydramnios in mid-trimester gestation that impedes the development of pulmonary vasculature in the fetus^15,16^.

The present study also confirmed that EPIs with early PH had a higher incidence of death and m/s BPD than those without. Whether the described term is “delayed cardiopulmonary adaptation”^4^, “early pulmonary vascular disease”^3^, “clinical PPHN”^13^, or even “hypoxic respiratory failure”^14^, early PH in preterm infants has been a definite risk factor for death or BPD in preterm infants^12,15^.

On the other hand, late PH occurs most prevalently in EPIs with established BPD. Reported incidence rates of late PH in preterm infants with established m/s BPD also vary, such as 44.4% by Bhat et al.^10^, 19.7% by Mourani^3^, and 27.7% by Weismann^17^ in prospectively designed studies, with 20% prevalence reported by a recent meta-analysis^18^, the latter of which is similar to our finding. A stricter definition by Mirza et al.^2^ showed a much lower incidence (8.3%) when they omitted the septal position for the definition of PH.

The prognosis of late PH is grave. Late PH significantly increases mortality in patients with BPD, as a recent meta-analysis has shown fivefold higher odds of mortality in BPD-associated PH compared to BPD alone^5^. In the present study, a fourfold increase in mortality (21% vs. 5.3%) after 36 weeks PMA and before discharge was observed in BPD-associated late PH compared to BPD alone.

Although late PH occurs in infants without BPD, the relationship between BPD and late PH is very strong, and independent risk factors of BPD-associated PH have not been clearly evaluated^6^. Lower birthweight, SGA^19^, severity of BPD^18^, ROP, and NEC^17^ were significant risk factors for BPD-associated PH. However, in the present study, neither birthweight, SGA, ROP, nor NEC were related to the development of late PH in BPD patients, while prolonged (> 28 days) ventilation was significantly related. A recent meta-analysis also revealed that the duration of ventilation is associated with the development of late PH^18^. Although we did not find a correlation between SGA or gestational age and late PH in the present study, it should be the focus of future large-scale studies as numerous studies have found an association^6,19,20^.

The dose-dependent response of the incidence of late PH with BPD severity indicates that the incidence of late PH is higher with increasing BPD severity^5,18^. Interestingly, in the present study, we observed that late PH developed with similar incidences in moderate and severe BPD patients. Previously, some reports showed no definite relationship between BPD severity and the development of late PH^21^. Thus, the association between BPD severity and the development of late PH needs to be clarified in further studies.

Whether early PH is an independent and significant risk factor for the development of late PH has been controversial. In one prospective study^3^, no clinical risk factors were found for late PH at 36 weeks PMA, except for 7-day echocardiographic findings of any septal flattening. However, in that study, late PH was seen in 9–10% in BPD-free or mild BPD patients, which comprised 39% of all late PH infants. Whether the risk factors for late PH occurring in BPD are different from those for late PH occurring in non-BPD have not been investigated. However, no other studies have demonstrated that early PH is a risk factor for late PH associated with BPD^2,7,8^. Therefore, the present study is the first to demonstrate a clear association between early PH and late PH in established BPD patients who were born as EPIs. In the present study, nearly half (45.2%) of the EPIs who showed early PH within 7 days of life and then survived until 36–38 weeks PMA with m/s BPD developed late PH. On the contrary, under one-fifth (14.5%) of EPIs who did not show any signs of early PH but survived with m/s BPD later, developed late PH. Among BPD infants, the degree of impact of the presence of early PH on the increased risk for BPD-associated late PH was similar to that of prolonged (> 28 days) invasive ventilation. These findings suggest that the pathogenesis of BPD-associated late PH might be strongly related to not only postnatal ventilator-induced lung injuries but also prior pulmonary vascular injury early in life.

In the present study, PDA was managed with conventional nonintervention treatment based on our unit policy^22^. Therefore, the effect of NSAID treatment for PDA in EPIs on the development of late PH could not be evaluated. Furthermore, NSAID treatment data for PDA were not available for 57 patients enrolled in a PDA trial comparing BPD incidence between infants with and without NSAID treatment^23^. However, the proportion of infants with and without early PH (Table 2) or late PH (Supplementary Table 4) enrolled in that trial was similar, and the NSAIDs were randomly and equally allocated. Therefore, the possibility that the NSAID treatment altered the risk of late PH in the present study is extremely low. However, whether NSAID treatment or any procedure intended to induce early ductal closure resulted in aggravated PH persistence or progression in infants with preexisting early PH was unanswered in the present study. The role of NSAID treatment for PDA in the development of late PH remains to be elucidated.

The present study has some limitations. Although late PH screening by echocardiography was prospectively performed as a routine screening to detect PH in established BPD patients, we could not confirm that early echocardiographic examinations on PND 4–7 focused on the detection of early PH because of the retrospective nature of this study, which could result in underestimation of the incidence of early PH. This was a single-center study with a small sample size, especially in the subgroup analysis, and without long-term follow-up data. Although the present study showed a strong association between early and late PH, especially in the subset cohort of preterm infants with m/s BPD, it is unknown if this observation is unique to BPD patients, if the effect is more powerful than in mild/non-BPD patients, or if there is an association between early and late PH observed in any random sampling of EPI. This is because infants with no or mild BPD did not have echocardiographic data at 36–38 weeks PMA; thus, it is unknown whether these infants developed late PH in the present study.

However, the strength of this study lies in the relatively large number of EPIs included with gestational age of 22–27 weeks. They were managed homogeneously in one center, and echocardiograms were available at 4–7 PND in all included EPIs. Moreover, echocardiograms were performed routinely to detect late PH in a narrow time window of 36–38 weeks PMA in all m/s BPD infants during the study period, which reduced potential bias.

In conclusion, Infants with early PH had a 6.6 times higher risk of death before 36 weeks PMA or BPD-PH than those without early PH, which was comparable to late sepsis showing a 6.7 times higher risk. Among infants with m/s BPD, early PH increased the risk of late PH by 4.5 times, which is also comparable to that of prolonged ventilator care (> 28 days), showing an increased risk by 4.9 times. Therefore, early PH detected by echocardiography in early life is an independent risk factor for the development of BPD-associated late PH in EPIs. When echocardiographic signs of early PH on PND 4–7 in EPIs of 22–27 weeks’ gestational age are observed, careful monitoring with counseling is needed not only to decrease the incidence of mortality and BPD but also to avoid the development of BPD-associated late PH. Furthermore, echocardiographic screening for early PH on PND 4–7 can be a useful tool for targeting or allocating a study population to develop safe therapeutic strategies for BPD-associated late PH patients.

## Methods

This retrospective study included infants born at and admitted to the Samsung Medical Center between 2012 and 2017 with gestational age of 22–27 weeks and birthweight of 401–999 g. Infants with congenital malformations (except for atrial septal defect, patent foramen ovale < 2 mm) and absent echocardiographic data on PND 4–7 were excluded from this study. During the study period, the EPIs underwent echocardiographic examination at around 1 week of life almost routinely for the evaluation of hemodynamically significant PDA or hemodynamic stability. We have previously shown that late PH was significantly associated with the m/s BPD^24^. Therefore, in 2012, our unit implemented a routine echocardiogram screening program to detect late PH in all infants diagnosed with m/s BPD between 36–38 weeks PMA. This enabled us to conduct prospective screening of late PH in every m/s BPD patient during the study period. PDA was managed using conventional nonintervention treatment (not to treat) with careful fluid management based on our unit policy^22^. During the study period, 57 infants were included in clinical trial comparing the BPD incidence between nonintervention or oral ibuprofen for PDA in preterm infants^23^. Data on ibuprofen treatment for PDA were not available because double blindness was maintained until long-term neurodevelopmental outcome data were obtained, and data collection was in progress (previously published data^23^ are the result of an interim analysis with prior approval). Data collection for the present study was approved by the Institutional Review Board of Samsung Medical Center, which waived the need for informed consent for the retrospective chart review (IRB 2018-02-085). The study was conducted in accordance with international and institutional guidelines for research involving human subjects, in accordance with the Declaration of Helsinki.

Echocardiography was performed at 4–7 PND after the usual transitional period using VividTM S60N (GE Healthcare, Chicago, IL)^9^. If infants were diagnosed with m/s BPD at 36 weeks PMA, echocardiography was performed according to the routine protocol within 2 weeks (36–38 weeks PMA). All echocardiography procedures were performed by pediatric echocardiographic specialists, pediatric cardiology fellows, or pediatric cardiologists during the study period. Whereas early echocardiography was done with a request by neonatologists and was not specially focused on PH, late echocardiography was performed routinely for PH screening in patients with m/s BPD within the time window of 36–38 weeks PMA. All images were reviewed and reported by two pediatric cardiologists at the time of the initial individual examination. In the present study, we retrospectively reviewed all echocardiograms to determine which patients had PH based on the initial clinical assessment of the cardiologist. PH was diagnosed and defined when the echocardiographic findings met at least one of the following criteria^9,25–27^: (1) systemic or suprasystemic pulmonary artery pressure (> 40 mmHg) as estimated by peak Doppler velocity of tricuspid regurgitation^26^, (2) right-to-left or bidirectional shunting of blood through the PDA, patent foramen ovale, or atrial septal defect; or (3) flattened interventricular septum or D-shaped left ventricle at end systole. We defined early PH as a diagnosis on 4–7 PND among all infants and late PH when diagnosed at 36–38 weeks PMA among m/s BPD infants.

For this study, we defined study exposure as early echocardiographic evidence of PH at 4–7 PND and primary outcome as death before 36 weeks PMA or late echocardiographic evidence of PH in m/s BPD infants.

Data on maternal and perinatal characteristics, neonatal demographics, diseases, and outcomes were collected. Maternal factors included maternal age, multiple gestation, pregnancy-induced hypertension, oligohydramnios, in vitro fertilization, chorioamnionitis, mode of delivery, gestational diabetes, antenatal steroids, and PPROM. Oligohydramnios was defined as an amniotic fluid index < 5 on fetal ultrasound before delivery. Clinical chorioamnionitis was assessed by maternal and fetal clinical symptoms including maternal fever (> 38 °C), leukocytosis, fetal tachycardia, tenderness on the uterus, or foul-smelling amniotic fluid. Pathologic chorioamnionitis was confirmed by placental biopsy. PPROM was defined as more than 24 h between rupture and onset of labor. Neonatal characteristics included gestational age, birthweight, SGA (< 10th percentile), Apgar scores at 1 and 5 min, and sex. Neonatal diseases and outcomes included IVH, periventricular leukomalacia (PVL), PDA, BPD, NEC, sepsis, ROP, and mortality. The definitions of the disease are as follows. IVH in cranial ultrasound was diagnosed and graded based on the criteria according to the classification of Papile et al.^28^. PVL was also diagnosed according to white matter changes using cranial ultrasound^29^. The diagnosis and severity of BPD were assessed using severity-based diagnostic criteria from the National Institutes of Health^30^. NEC was diagnosed and graded according to Bell's criteria^31^. Sepsis was defined as proven in the blood culture. According to the time of onset, sepsis was divided into early sepsis within 7 days of birth and late sepsis after 7 days. Mortality was confirmed as death prior to discharge. ROP was diagnosed using the International Classification for ROP by an ophthalmologist^32^.

Demographic characteristics and outcomes were compared between EPIs with and without early PH. EPIs with and without primary outcome, death before 36 weeks PMA or m/s BPD with late PH, were then compared. Descriptive statistics for continuous variables are reported as mean and standard deviation. Categorical variables are reported as frequencies and percentages. Comparisons of the groups’ demographic factors and outcomes were performed using the t-test. χ^2^ or Fisher’s exact tests for proportions were performed. A logistic regression model was performed to determine the risk factors for late PH. Risk factors for primary outcome were evaluated by odds ratios and confidence intervals. We analyzed the risk factors for primary outcome with gestational age and risk factors that were significantly different between those with and without primary outcome (*P* < 0.05) by multivariate logistic regression analyses. All statistical analyses were performed using SPSS version 25 (IBM, Armonk, NY) and *P*-values < 0.05 were considered statistically significant.

## Supplementary Information

Supplementary Tables.
