# Supplementary material for: Early pulmonary hypertension is a risk factor for bronchopulmonary dysplasia-associated late pulmonary hypertension in extremely preterm infants
Source: Sci Rep. 2021 May 27;11:11206. doi: 10.1038/s41598-021-90769-4 (PMC8160152; doi:10.1038/s41598-021-90769-4)
Supplement: Supplementary file 1 — Supplementary Tables. [file 41598_2021_90769_MOESM1_ESM.pdf]

Early pulmonary hypertension is a risk factor for bronchopulmonary dysplasia-associated late pulmonary hypertension in extremely preterm infants

Hyun Ho Kim,[1,2#] Se In Sung,[3#] Mi Sun Yang,[3] Yea Seul Han,[4] Hye Seon Kim,[3] So Yoon Ahn,[3] Ga Won Jeon,[5] Yun Sil Chang,[3*] Won Soon Park[3]

[#] Co-first authors, [*] Corresponding author

[1] Research Institute of Clinical Medicine of Jeonbuk National University, Jeonju, Korea

[2] Biomedical Research Institute of Jeonbuk National University Hospital, Jeonbuk National University School of Medicine, Jeonju, Korea

[3] Department of Pediatrics, Samsung Medical Center Sungkyunkwan University School of Medicine, Seoul, Korea

[4] Department of Pediatrics, Hallym University Kangnam Sacred Heart Hospital, Seoul, Korea

[5] Department of Pediatrics, Busan Paik Hospital, Inje University College of Medicine, Busan, Korea

Corresponding Author

Yun Sil Chang, MD, PhD

Department of Pediatrics, Samsung Medical Center Sungkyunkwan University School of Medicine
81 Irwon-Ro, Gangnam-Ku, Seoul 06351, Korea

Tel: +82-2-3410-3528, 3539

Fax: +82-2-3410-0043

Email: yschang@skku.edu

Supplemental Table 1. Diagnosis and echocardiographic findings of early and late PH

	**Early PH (N=74)**	**Late PH (N=23)**
SPAP>40, n (%)	9 (12.2%)	6 (26.1%)
SHUNT, n (%)	0 (0.0%)	0 (0.0%)
IVS, n (%)	49 (66.2%)	9 (39.1%)
SPAP>40 and SHUNT, n (%)	0 (0.0%)	0 (0.0%)
SPAP>40 and IVS, n (%)	13 (17.6%)	8 (37.8%)
SHUNT and IVS, n (%)	2 (2.7%)	0 (0.0%)
SPAP>40, SHUNT, and IVS, n (%)	1 (1.4%)	0 (0.0%)

PH, pulmonary hypertension; SPAP>40, systemic or suprasystemic pulmonary artery pressure >40 mmHg as estimated by peak Doppler velocity of tricuspid regurgitation; SHUNT; right-to-left or bidirectional shunting of blood through patent ductus arteriosus, patent foramen ovale, or atrial septal defect; IVS, flattened interventricular septum or D-shaped left ventricle at end systole. N(%) or mean (SD).

Supplement Table 2. Perinatal demographic characteristics among subgroup of moderate to severe BPD patients

	No BPD-PH (N=70)	BPD-PH (N=23)	*P* value
**Neonatal characteristics**			
Gestational age, (week)	25.0±1.2	25.1±1.3	0.61
22+0 to 23+6 weeks, n (%)	15 (21.4)	4 (17.4)	0.68
24+0 to 25+6 weeks, n (%)	40 (57.1)	12 (52.2)	0.68
26+0 to 27+6 weeks, n (%)	15 (21.4)	7 (30.4)	0.38
Birthweight (g)	709.7±124.5	663.0±178.1	0.17
400-600 g, n (%)	13 (18.6)	8 (34.8)	0.11
600-800 g, n (%)	38 (54.3)	9 (39.1)	0.16
800-1000 g, n (%)	19 (27.1)	6 (26.1)	0.92
Small for gestational age, n (%)	12 (17.1)	9 (39.1)	<0.05
Male, n (%)	39 (55.7)	12 (52.2)	0.77
1-min Apgar score	4.3±1.3	4.0±1.6	0.41
5-min Apgar score	7.0±1.1	6.7±1.5	0.29
Age at echocardiography, postnatal days	84.1±10.9	85.1±10.5	0.70
PMA at echocardiography, weeks	36.9±0.7	37.2±0.9	0.18
**Maternal characteristics**			
Maternal age, year	33.0±4.0	33.9±3.9	0.37
Multiple gestation, n (%)	24 (34.3)	10 (43.5)	0.43
Pregnancy induced hypertension, n (%)	8 (11.4)	4 (17.4)	0.48
Oligohydramnios, n (%)	19 (27.1)	10 (43.5)	0.14
In vitro fertilization, n (%)	21 (30.0)	9 (39.1)	0.42
Clinical chorioamnionitis, n (%)	38 (54.3)	13 (56.5)	0.85
Pathologic chorioamnionitis, n (%)	48 (68.6)	15 (65.2)	0.77
Cesarean section, n (%)	53 (75.7)	16 (69.6)	0.56
Gestational diabetes, n (%)	4 (5.7)	0 (0.0)	0.57
Complete antenatal steroid, n (%)	43 (61.4)	17 (73.9)	0.28
Duration of PPROM, day	4.9±9.3	5.9±10.2	0.69
PPROM duration ≥28 days, n (%)	1 (1.4)	3 (13.0)	<0.05
PPROM at <26+0 weeks, n (%)	9 (12.9)	3 (13.0)	1.00

PH, pulmonary hypertension; BPD, bronchopulmonary dysplasia, PPROM, Preterm premature rupture of membranes; PMA, post-menstrual age. Data are show as N (%) or mean±SD.

Supplemental Table 3. Main cause of death before 36 weeks PMA

	All (N=39)	No Early PH (N=18)	Early PH (N=21)	*P* value
IVH, n (%)	3 (7.7)	1 (5.6)	2 (9.5)	1.00
Pneumonia, n (%)	2 (5.1)	2 (11.1)	0 (0.0)	0.21
Pulmonary hypoplasia, n (%)	6 (10.3)	0 (0.0)	6 (28.6)	<0.05
Pneumothorax, n (%)	1 (2.6)	0 (0.0)	1 (4.8)	1.00
NEC, n (%)	12 (30.8)	6 (33.3)	6 (28.6)	0.75
Intestinal perforation, n (%)	2 (5.1)	2 (11.1)	0 (0.0)	0.21
Sepsis, n (%)	13 (33.3)	7 (38.9)	6 (28.6)	0.31

PMA, Post-menstrual age; PH, Pulmonary hypertension; IVH, Intraventricular hemorrhage; NEC, Necrotizing enterocolitis, Data are show as N (%)

Supplement Table 4. Neonatal morbidities and mortality among moderate to severe BPD

	No late PH (N=70)	Late PH (N=23)	*P* value
Early PH, n (%)	17 (24.3)	14 (60.9)	<0.05
IVH grade 3-4, n (%)	9 (12.9)	2 (8.7)	0.73
PDA >2mm (%) at early echocardiogram*	45 (64.3)	18 (78.3)	0.21
Enrollment for PDA RCT [23], (%)	23 (32.9)	8 (34.8)	0.87
NEC grade 2b-3, n (%)	7 (10.0)	2 (8.7%)	1.00
Invasive ventilator before 36 weeks PMA, day	43.7±23.3	51.4±22.1	0.17
Invasive ventilator before 36 weeks PMA >28 days, n (%)	46 (65.7)	21 (91.3)	<0.05
Early sepsis (<7 days), n (%)	2 (2.9)	1 (4.3)	1.00
Late sepsis (7 days ~ 36 weeks PMA), n (%)	13 (18.6)	8 (34.8)	0.11
ROP stage 3-4, n (%)	44 (62.5)	15 (65.2)	1.00
Periventricular leukomalacia, n (%)	7 (10.0)	5 (21.7)	0.16
Death after 36 weeks PMA, n (%)	4 (5.7)	5 (21.7)	<0.05

*PDA was managed using conventional nonintervention treatment (not to treat) with careful fluid management based on our unit policy [22]; BPD, bronchopulmonary dysplasia; PH, Pulmonary hypertension; IVH, Intraventricular hemorrhage; PDA, Patent ductus arteriosus; Randomized clinical study, NEC, Necrotizing enterocolitis; PMA, Post-menstrual age; ROP, Retinopathy of premature, Data are show as N (%) or mean±SD
